# An Innovative Bio-Vehicle for Resveratrol and Tocopherol Based on Quinoa 11S Globulin—Nanocomplex Design and Characterization

**DOI:** 10.3390/pharmaceutics16091118

**Published:** 2024-08-24

**Authors:** Alejandra J. Rubinstein, Guadalupe Garcia Liñares, Valeria Boeris, Oscar E. Pérez

**Affiliations:** 1 Consejo Nacional de Investigación Científica y Técnicas de la República Argentina, IQUIBICEN-CONICET, Departamento de Química Biológica, Facultad de Ciencias Exactas y Naturales, Universidad de Buenos Aires, Intendente Güiraldes, s/n, Ciudad Universitaria, Buenos Aires C1428EGA, Argentina; arubinstein@qb.fcen.uba.ar; 2 Laboratorio de Biocatálisis, Departamento de Química Orgánica y UMYMFOR, Facultad de Ciencias Exactas y Naturales, Universidad de Buenos Aires-CONICET, Intendente Güiraldes, s/n, Ciudad Universitaria, Buenos Aires C1428EGA, Argentina; linares@qo.fcen.uba.ar; 3 Área Fisicoquímica, Departamento de Química Física, Facultad de Ciencias Bioquímicas y Farmacéuticas, Universidad Nacional de Rosario (UNR)—CONICET, Suipacha 531, Rosario S2002LRK, Argentina; valeriaboeris@conicet.gov.ar

**Keywords:** 11S globulin, quinoa, bioactive compounds, complexation, aggregation, nutraceutical products

## Abstract

Nanocomplexes, which possess immense potential to function as nanovehicles, can link diverse ligand compounds. The objective of the present study was to design and characterize resveratrol (RSV)- and tocopherol (TOC)-loaded 11S quinoa seed protein nanocomplexes. Firstly, molecular docking was performed to describe the probable binding sites between protein and ligands, and binding energies of −5.6 and −6.2 kcal/mol were found for RSV and TOC, respectively. Isothermal titration calorimetry allowed us to obtain the thermodynamic parameters that described the molecular interactions between RSV or TOC with the protein, finding the complexation process to be exothermic and spontaneous. 11S globulin intrinsic fluorescence spectra showed quenching effects exerted by RSV and TOC, demonstrating protein–bioactive compound interactions. The application of Stern–Volmer, Scatchard, and Förster resonance energy transfer models confirmed static quenching and allowed us to obtain parameters that described the 11S-RSV and 11S-TOC complexation processes. RSV has a higher tendency to bind 11S globulin according to ITC and fluorescence analysis. Secondly, the protein aggregation induced by bioactive compound interactions was confirmed by dynamic light scattering and atomic force microscopy, with diameters <150 nm detected by both techniques. Finally, it was found that the antioxidant capacity of a single 11S globulin did not decrease; meanwhile, it was additive for 11S-RSV. These nanocomplexes could constitute a real platform for the design of nutraceutical products.

## 1. Introduction

Quinoa (*Chenopodium quinoa* Willd.) is an ancient crop indigenous to the Andean regions of South America. In recent years, quinoa seeds have gained renewed attention due to their exceptional nutritional properties, including high-value proteins and the presence of antioxidant molecules [1,2]. Quinoa’s 11S globulin, also known as chenopodin, represents the most significant seed storage protein, and has a structure resembling glycinin, the 11S globulin found in soybeans. Quinoa 11S globulin is composed of a basic subunit of 17–20 kDa and an acid subunit of 30–35 kDa interconnected by disulfide bonds [3]. Resveratrol (RSV) (3,5,4′-trihydroxystilbene) is categorized as a non-flavonoid polyphenol, naturally occurring in both trans and cis isomers. Numerous studies have demonstrated the efficacy of RSV in mitigating a wide range of diseases, including diabetes mellitus, metabolic syndrome, obesity, inflammation, cardiovascular issues, and neurodegenerative conditions [4]. The questions concerning the biological activity, sources, and methods of analysis were very recently reviewed and critically discussed in detail [5]. However, RSV has low water solubility and limited bioavailability. Additionally, RSV is highly vulnerable to oxidative conditions, leading to rapid degradation and extensive metabolism. Therefore, the constrained bioavailability underscores the necessity for the development of more suitable RSV formulations to protect its bioactivity [6]. α-Tocopherol (TOC) represents the predominant and biologically active variant of lipophilic vitamin E, known for its capacity to diminish the risk of various chronic diseases linked to oxidative stress. Numerous studies have demonstrated the potential health benefits associated with the consumption of vitamin E. However, the application of this nutrient is complicated by its hydrophobic nature and its inherent sensitivity to oxygen, heat, and light. Tocopherols are easily oxidized when exposed to air, especially in the presence of iron [7].

Protein molecules have the capacity to spontaneously link ligands. In this context, previous reports have indicated this phenomenon as a strategy to transport bioactive compounds of importance to public health, thus constituting true functional ingredients [8,9,10,11,12,13]. Specifically, when a bioactive compound interacts with a protein, a novel structure emerges, the complex, which could form aggregates via self-assembly. Consequently, self-assembled complexes constitute nanocomplexes when their dimensions are on a nanoscale [14]. Nanocomplexes, which possess immense potential to function as nanovehicles, can link diverse ligand compounds. One particularly intriguing characteristic of these nanocomplexes is their ability to encapsulate and release preloaded compounds, such as RSV or TOC, within a specific environment. The release mechanism of the bioactive compound, the ligand, can be regulated through adjustments in pH levels, temperature, or ionic strength [15].

Various endeavors have been undertaken to create nanovehicles through nanocomplexation systems, enabling the targeted release of biologically significant compounds in the pharmaceutical, nutraceutical, and food industries. In this context, the aim of this study was to design and characterize quinoa seed 11S globulin-based nanocomplexes containing RSV or TOC. The underlying idea for this approach is to generate vehicles for RSV and TOC, which can be protected from insulting agents and, in turn, allow the possibility of exerting controlled release under specific conditions, constituting true functional ingredients for nutraceutical or functional products. The objective of this work was to generate quinoa 11S globulin-based complexes containing RSV and TOC, as well as to characterize these entities in terms of their physicochemical, structural, and antioxidant properties.

## 2. Materials and Methods

### 2.1. Materials

Defatted quinoa (*Chenopodium quinoa* Willd.) seed flour was obtained from “El Portugues” (seed origin: Peru); the protein content was 16% db. The protein fraction, in a yield of 60%, was isolated from the defatted quinoa seed flour according to the method proposed by Martinez et al. [12]. Briefly, 10 g of defatted flour was stirred into distilled water at pH 9 for 1 h at 20 °C. Then, the albumin fraction was removed by centrifugation at 10,000× *g* for 10 min (RC-5 Superspeed refrigerated centrifuge, Sorval, Minneapolis, MN, USA). This first pellet was discarded, and the supernatant was adjusted to pH 4.5 with HCl 0.1 M and centrifuged under the same conditions. This second pellet, enriched in 11S, was resuspended into distilled water at pH 9 and centrifuged as before. The resulting pellet was discarded and the third supernatant, containing mainly the native 11S globulin, was centrifuged under the same conditions and then submitted to a chromatography separation process (see details in Section 2.2). Protein determinations were performed using the Kjedahl method (Nx6.25). TOC and RSV were generously donated by DSM and Temis Lostaló, respectively, both laboratories from Argentina, and used without further purification. Milli-Q water was always used, and all chemicals were of analytical grade.

### 2.2. 11S Purification

An ÄKTA Protein Purification System, FPLC (Fast Protein Liquid Chromatography), was used for quinoa seed 11S globulin purification (GE Healthcare Life Sciences, Freiburg, Germany). It was equipped with a Sephadex^®^ S200 10/300 GL (GE Healthcare Life Sciences, Uppsala, Sweden) column. Aliquots of 1 mL containing the total globulins equilibrated for 12 h at 20 °C were submitted to the size exclusion chromatography process. Samples were eluted with distilled water at pH 9, at a flow rate of 0.3 mL/min at 25 °C. Fractions of 1.5 mL were collected and analyzed by absorbance at 280 nm. The eluted fraction corresponding to 11S globulin was stored at −20 °C until further use.

### 2.3. SDS-PAGE Electrophoresis

The fraction corresponding to total globulins was analyzed in SDS-PAGE according to Laemmli [16] with a Mini-Protean II device (Bio-Rad, Hercules, CA, USA). A 12% polyacrylamide running gel was used under denaturing conditions. Runs were performed at 90 V, and 40 µg of protein was deposited in each well of the stacking gel. After electrophoresis, Coomassie brilliant blue staining was performed to detect proteins of interest [17].

### 2.4. Molecular Docking

The approach was carried out using AutoDock vina 1.1.2 [18]. Given the absence of a crystal structure for quinoa seed 11S globulin, modeling was performed using the 11S globulin of *Amaranthus hypochondriacus* (pdb: 3qac) as a homologous structure. Using Blastp against the Protein Data Bank (PDB) platform, we established BLOSUM62 as the matrix, with the following parameters: gap cost of 11; extension 1 and word size of 6. The crystallized structures of 11S proteins present in *Amaranthus hypochondriacus*, *Bertholletia excelsa*, *Pisum sativum*, *Prunus dulcis* and *Cocos nucifera* yielded the best homologous results, with a maximum score of 43.85% and an e-value of 1.10^−115^ [19]. Through the use of Pfam (Protein family), it was shown that the Cupin 1 domain was the common motif of the set of proteins, with an average bit-score value of 104.3 and an average e-value of 4.5 × 10^−26^ [20].

TOC and RSV conformers were obtained from PubChem (https://pubchem.ncbi.nlm.nih.gov) (accessed on 6 December 2022) as sdf files, which were transformed into mol2 using the Avogadro 1.2.0 molecular editor. The docking analysis was performed based on the protocolos outlined by Bustos et al. and Morris et al. [21,22]. Briefly, blind dockings with a fixed receptor of all protein–bioactive pairs were performed, with grids that covered the entire macromolecule. To ensure the reproducibility of the results, all dockings were repeated 10 times. The lowest energy pose of each complex was analyzed using the VMD v.1.9.3 (visual molecular dynamics) [23], Ligplot and PLIP (protein ligand interaction profile) programs.

### 2.5. Preparation of 11S and TOC or RSV Mixed Systems

Samples of 11S globulin and RSV or TOC were dissolved separately in the appropriate solvent and then diluted into distilled water at pH 9 at room temperature under gentle agitation. TOC was prepared in a stock solution of 10% in 96% ethanol. RSV was prepared by dissolving 0.00456 g in 1600 µL of 96% ethanol. This was then diluted to 4 mL with double-distilled water, yielding a 5 mM stock solution. The percentage of ethanol in mixed systems could be calculated. For instance, in the case of the highest concentrations of bioactive compounds, the ethanol concentration was 0.3% and 0.01% for the RSV and TOC solutions, respectively. Additionally, ethanol has a vapor pressure (Pv) of 50 mmHg at 25 °C, which is a moderate Pv value, higher than water (Pv water = 25 mmHg at 25 °C), indicating the higher possibility of solvent evaporation. Therefore, one can assume that the percentage of the remaining ethanol was very low, in order to affect the protein’s integrity.

The solutions were prepared freshly and centrifuged at 10,000 rpm for 10 min (Gyrospin, Labtech, Sorisole, Italy). The supernatant was kept at 4 °C for 24 h to achieve the complete hydration of the molecules. Thus, the final protein concentration of mixed systems was kept constant at 0.5%, *w*/*w*, while the RSV and TOC concentrations ranged from 0 to 6250 µM.

### 2.6. Circular Dichroism (CD)

For these experiments, single 11S and 11S-RSV and 11S-TOC mixed systems were diluted up to 0.02% *w*/*w* of protein with 10 µM of RSV and 250 µM of TOC, respectively. The CD spectrum was obtained using a circular dichroism spectrometer (JASCO J-815 CD spectrometer, Silver Spring, MD, USA). The wavelength ranged from 190 nm to 260 nm. The bandwidth was set at 1 nm. The spectra for each single bioactive compound, RSV or TOC, were also assessed, without obtaining significant signals. The percentage of protein secondary structure was calculated using the BestSel deconvolution software (https://bestsel.elte.hu/index.php (accessed on 10 July 2024)).

### 2.7. Isothermal Titration Calorimetry (ITC)

An ITC apparatus NanoITC (TA Instruments, New Castle DE, USA) was used to characterize the interactions between quinoa 11S globulin and RSV or TOC from a thermodynamic point of view. To this end, 11S globulin (36.4 μM) and RSV or TOC (876 μM) solutions were prepared using distilled water at pH 9. In the experiment, 50 μL RSV or TOC solution was gradually injected into 200 μL of 11S solution with the injection volume of 2.5 μL 20 times. The interval, stirring speed and temperature were set at 300 s, 300 rpm and 25 °C, respectively. To subtract the effect of dilution heat, the hydrophobic compound (RSV or TOC) solution was titrated into distilled water at pH 9, and the result was set as a blank. A series of thermodynamic parameters, including Gibbs free energy (ΔG), enthalpy change (ΔH), entropy change (ΔS) and the binding constant (Ka), were analyzed by the Nano Analyze software 2.1.

### 2.8. Steady-State Fluorescence Measurements

Fluorescence spectra for single 11S globulin and 11S-TOC or 11S-RSV mixed solutions were determined using a Cary Eclipse fluorescence spectrophotometer (ThermoSpectronic AMINCO-Bowman, Series 2, Madison, WI, USA) at 25 °C. Protein intrinsic fluorescence emission spectra were recorded from 290 to 400 nm with an excitation wavelength of 280 nm. Thus, RSV solutions with concentrations ranging from 0 to 25 µM and TOC ranging from 0 to 313 µM were evaluated in mixed solutions. Bioactive compounds fluorescence was also determined under that wavelength range to discard any possible emission. Intermolecular interactions of 11S and bioactive compounds were evaluated from the fluorescence maximum peak of each emission spectra corresponding to mixed solutions in comparison to the single 11S spectrum. The Stern–Volmer model can be employed to examine the relationship between fluorescence intensity and concentration dependence [24]:

(1) $$\frac{F_{0}}{F}=1+k_{q}\times \tau_{0}\times \left[BIO\right]=1+K_{SV}\times [BIO]$$

where [*BIO*] is the concentration of the bioactive compound acting as a quencher; *F*_0_ and *F* are the fluorescence emission intensities with and without the quencher, respectively; *kq* is the fluorescence quenching rate constant; *τ*_0_ is the fluorescence lifetime of the fluorophore in the absence of the quencher; and *K_SV_* is the Stern–Volmer quenching constant. A linear plot of *F*_0_*/F* as a function of [*BIO*] allowed us to obtain the *Ksv* values from the slope of the straight line. *τ*_0_ was reported to be equal to 2.9 ns for the Trp residues of 11S [12,25].

When small molecules independently bind to a group of identical sites on a macromolecule, the equilibrium between unbound and bound molecules can be described using the equation introduced by Bian [26]:

(2) $$log(\frac{F_{0}-F}{F})=log(K_{a})+n\times log\left[BIO\right]$$

where *Ka* and *n* are the apparent binding constant and the number of binding sites per 11S molecule, respectively. From the intercept and slope of *log (F*_0_ − *F)/F* vs. *log* [*BIO*], the values adopted by *Ka* and *n* can be obtained. The *Ka* value indicates the magnitude of the interaction between the protein and ligand. Furthermore, the *n* parameter value indicates the sites of association on the protein molecule [24].

The model described by Scatchard (Equation (3) and (4)), as detailed by Wei et al. [27], represents an alternative mathematical approach to that used here to analyze the binding phenomena between 11S globulin and TOC or RSV from the fluorescence experimental data.

(3) $$\left[11S\right]\times \left(1-f_{i}\right)=\frac{\left[BIO\right]}{n*\left(\frac{1}{f_{i}}-1\right)}-\frac{1}{n*K_{s}}$$

(4) $$f_{i}=\frac{f_{li}-f_{lo}}{f_{lmax}-f_{lo}}$$

where *f_li_* is the maximal intensity in each measured spectrum, *f_lo_* is the intensity without quencher (bioactive compound) and *f_lmax_* is the intensity with the highest concentration of quencher; [*11S*] is the protein concentration and [*BIO*] is the bioactive compound concentration; *Ks* and *n* are the apparent binding constant and the number of binding sites per 11S molecule, respectively.

### 2.9. Encapsulation Efficiency

The amount of RSV or TOC bound to 11S globulin was determined by the difference between the concentration of the bioactive compound initially added to the mixed solutions *(BIO_N_)* minus the bioactive compound not bound or free *(BIO_S_)* [13]. This further refers to the amount of RSV or TOC in the supernatant after ultracentrifugation and filtration through a 10 kDa cut off unit (Vivaspin Turbo 15, Sartorius, Gloucestershire, UK). Here, 5 mL of each sample, 11S-TOC or 11S-RSV 0.1%, in distilled water at pH 9 were centrifuged in the filters. The bioactive compound solution was used as a control to determine any loss due to binding to the filter unit. Filters were centrifuged at 4500× *g* for 15 min at 24 °C (RC-5 Superspeed refrigerated centrifuge, Sorval, Minneapolis, MN, USA). The flow through was collected and RSV or TOC concentration was determined. For RSV, absorbance at 306 nm was measured at 25 ± 1 °C on a V-570 UV-visible Jasco spectrophotometer (Tokyo, Japan). TOC was measured according to the Demirkaya and Kadioglu [28] method with an Agilent 7820A chromatographer equipped with a FID detector and G4513A autosampler. Then, the EE (%) of RSV and TOC for 11S globulin inspired complexes was determined as:

(5) $$EE\left(\%\right)=\frac{{BIO}_{N}-{BIO}_{s}}{{BIO}_{N}}\times 100$$

### 2.10. Particle Size and ζ-Potential Determinations

Dynamic light scattering (DLS) experiments were conducted using a Horiba Scientific nanoPartica Z-100 (Kyoto, Japan) apparatus. The measurements were carried out at 25 °C. Samples were contained in a Hellma quartz glass cuvette. The analysis of intensity fluctuations provided the diffusion coefficient of the particles, allowing the determination of particle size through the Stokes–Einstein equation. The interpretation of results followed the approach outlined in Perez et al. [15]. For the DLS analysis, concentrations of RSV in mixed solutions ranged from 10 to 50 µM, while TOC concentration ranged from 250 to 1500 µM.

Additionally, ζ–potential measurements were performed using the same DLS instrument. Samples were contained in semi-disposable carbon cells. The ζ–potential was derived from the electrophoretic mobility of the particles, and the conversion of these data into ζ–potential was accomplished using Henry’s equation.

### 2.11. Atomic Force Microscopy (AFM)

The methodology used by Carpineti et al. [29] was followed. Briefly, 5 µL of each suspension, 0.05% *w*/*w*, was put on a freshly cleaved muscovite mica. Images were taken in an atmosphere dried under a gentle stream of filtered, dry nitrogen. The cantilever used was an MPP-11100 from NanoDevices with a length of 125 μm and an elastic constant of 40 N/m, a resonance frequency of 300 Khz, and a tip radius of 8 nm. All images that are shown were analyzed by tapping mode in air. Nanoscope Software v1.40r1 was used to process the images by flattening to remove the background slope. Experiments were carried out in a temperature-controlled room at 20 ± 1 °C, with acoustic hood isolation and active vibration damping.

### 2.12. Antioxidant Activity of 11S-RSV or 11S-TOC Complexes

#### 2.12.1. ABTS Assay

The antioxidant capacities of 11S-RSV and 11S-TOC nanocomplexes were assessed through the ABTS assay, as outlined in Martinez et al. [12]. In this method, the pre-formed radical monocation of 2,2-azinobis-(3-ethylbenzothiazoline-6-sulfonic acid) (ABTS•+), obtained by oxidizing ABTS with potassium persulfate, serves as a marker. To initiate the process, ABTS was dissolved in water to achieve a final concentration of 7 mM. The ABTS•+ radical cation was generated by reacting the ABTS stock solution with a final concentration of potassium persulfate of 2.45 mM. The mixture was allowed to stand in darkness at room temperature for 12–16 h before use. The assessment of antioxidant activity for 11S-RSV or 11S-TOC nanocomplexes was conducted after diluting the ABTS•+ solution with phosphate buffer to achieve an absorbance of 0.80–0.90 (*A*_0_) at 734 nm (V-570, UV–visible Jasco spectrophotometer, Tokyo, Japan). Next, 2.5 mL of this solution was mixed with 0.5 mL of sample to finally measure the absorbance decrease (*A_inf_*). Therefore, ABTS•+ scavenging capacity was calculated as:

(6) $${ABTS•}^{+}\;scavenging\;capacity(\%)=\left(\frac{A_{0}-A_{inf}}{A_{0}}\right)\times 100$$

#### 2.12.2. Ferric Reducing Antioxidant Power (FRAP) Assay

The FRAP assay was executed following the methodology outlined by Martinez et al. [12]. Aliquots of 40 μL of 11S, RSV, TOC, 11S-RSV, or 11S-TOC nanocomplexes, along with the standard solution of GA, were dispensed into a test tube containing 600 μL of the FRAP reagent solution. The mixture was then kept in the dark at 25 °C for 30 min and the absorbance was measured at 593 nm (V-570, UV–visible Jasco spectrophotometer, Tokyo, Japan).

### 2.13. Statistical Analysis

All experiments were performed at least in triplicate. The results have been expressed as mean ± SD. The model’s goodness-of-fit was evaluated by the coefficient of determination (R^2^) using GraphPad Prism 8.0. software.

## 3. Results

### 3.1. SDS-PAGE- FPLC

**Figure 1:**
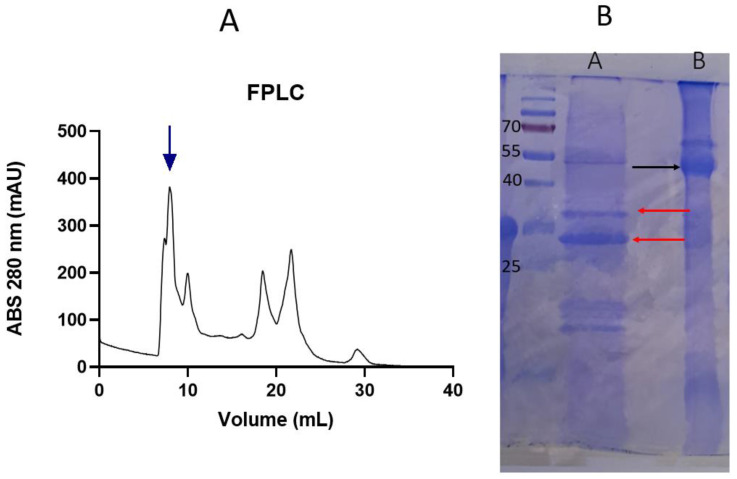
(**A**)—Elution profile by FPLC of quinoa protein extract. (**B**)—SDS-PAGE. Lane A: quinoa extract under reducing conditions. Red arrows: 11S basic and acid subunits. Lane B: Quinoa extract under non-reducing conditions. Black arrow: 11S (numbers indicate the MW of markers).

Size exclusion chromatography was utilized to isolate the quinoa 11S globulin fraction. The chromatograms initially showed a peak corresponding to a high-molecular-weight (MW) protein fraction and other peaks corresponding to smaller polypeptides (Figure 1A). The chromatogram pattern displayed various populations similar to protein extracts from quinoa, wheat, and whey, as reported previously [12,30,31]. Subsequently, SDS-PAGE was conducted to analyze the identity of the highest MW protein. The major protein band had a molar mass of approximately 55 kDa, indicating 11S. Due to the presence of disulfide bridges linking the acidic and basic subunits of quinoa 11S globulin, it was expected that, under non-reducing conditions, the subunits would migrate together (Figure 1B, lane B), while in the presence of β-mercaptoethanol, the 11S subunits would migrate separately (Figure 1B, lane A) [32]. Under non-reducing conditions, electrophoresis revealed the typical pattern of 11S globulin with acidic and/or basic subunits at 30–35 kDa and 17–20 kDa, respectively (Lane A). This pattern was previously observed by Brinegar and Goundan [33], who published the first report on the purification of 11S globulin from quinoa. Additionally, this pattern was also identified in the 11S globulins of other seeds, such as amaranth or soybeans. Abugoch et al. [34] investigated the isolation of amaranth proteins and discovered that the 11S globulin fraction consisted of two subunits with approximately 20 and 30 kDa, respectively.

### 3.2. Molecular Docking

**Figure 2:**
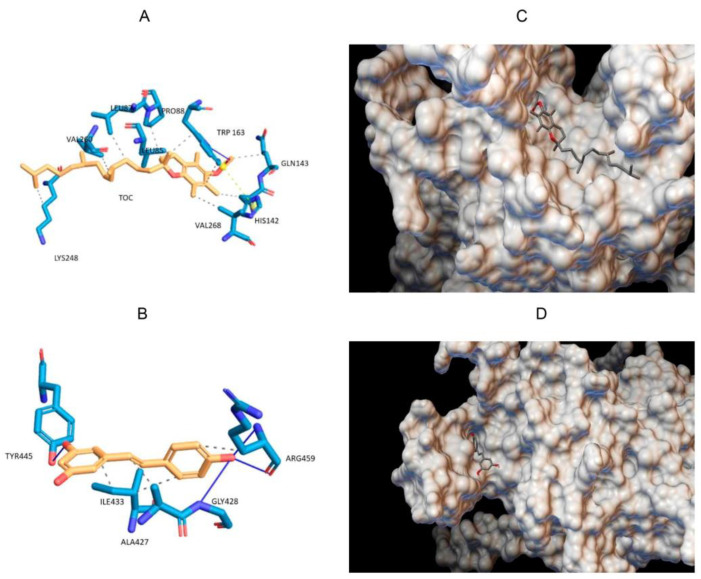
Binding site and mode with the lower energy poses for the systems consisting of TOC (**A**) and RSV (**B**), pointing out the amino acid residues involved in the binding with the analyzed bioactive compounds; the crystalline structure of 11S-RSV (**C**) and 11S-TOC (**D**), obtained by molecular docking.

Bioinformatics tools are useful for predicting the binding site and energy between a protein and a ligand. In this case the approach was used for quinoa 11S globulin with TOC or RSV. Docking is widely used to compare binding energies between different ligands or proteins [10,35,36]. The binding site and mode with the lowest energy of the TOC molecule into the quinoa 11S crystals is presented in Figure 2A. It is observed that the TOC bound and fit in the hydrophobic pocket formed by the amino acids LEU85, LEU87, PRO88, HIS142, GLN143, TRP163, LYS248 and VAL260 with a binding energy of −6.2 kcal/mol. This energy was the result of the formation of a hydrogen bond between the carbonyl oxygen of TOC molecule and the amino acid TRP163, a salt bridge, and several hydrophobic interactions, as seen in Table 1.

Docking was also performed between quinoa 11S globulin and RSV. The binding site and mode with the lowest energy pose for RSV in the 11S crystals are shown in Figure 2B. It can be observed that RSV bound the helices determined by the amino acids ALA427, ILE433 and ARG459 with a binding energy of −5.6 kcal/mol. This energy was the result of the formation of hydrogen bonds with the amino acids GLY428, TYR445 and ARG459, along with contributing several hydrophobic interactions, as seen in Table 1. The values of binding energy were very similar, showing no statistical differences between both bioactive compounds.

### 3.3. Circular Dichroism (CD)

**Figure 3:**
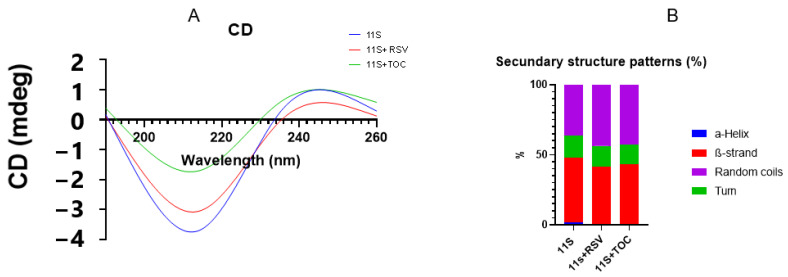
(**A**) Circular dichroism spectra and (**B**) secondary structure fractions for 11S globulin 0.02% and for 11S-RSV (0.02%-10 µM RSV) and 11S-TOC (0.02%-250 µM TOC) mixed systems.

To elucidate any changes induced by the bioactive compounds on the secondary structure of the 11S globulin, CD spectroscopy of the 11S-RSV and the 11S-TOC mixtures was performed. The mixed systems were prepared according to Section 2.5, using a final concentration of 0.02% 11S, 10 µM RSV, and 250 µM TOC. In general terms, the CD spectrum of 11S had a broad negative peak at 205 nm (Figure 3A). When the bioactive compounds were added, some changes were observed in the CD spectra of 11S, suggesting that the bioactive compounds altered the protein secondary structure, as explained by Liu et al. [11]. Specifically, Figure 3B shows that the secondary structure composition of single 11S globulin was 1.8% α-helix, 46% β-sheet, 15.9% β-turn and 36.3% other disordered patterns. When RSV was added, the contents of α-helix, β-sheet and β-turn were decreased to 0%, 41.6% and 14.7%, respectively, while the contents of other patterns were increased to 43.7%. When TOC was present in the system, the contents of α-helix, β-sheet and β-turn also decreased. In fact, for TOC, the CD peak shifted from 205 to 201 nm, suggesting that the conformation of 11S globulin transitioned from disorder to order [11].

### 3.4. Isothermal Titration Calorimetry (ITC)

**Figure 4:**
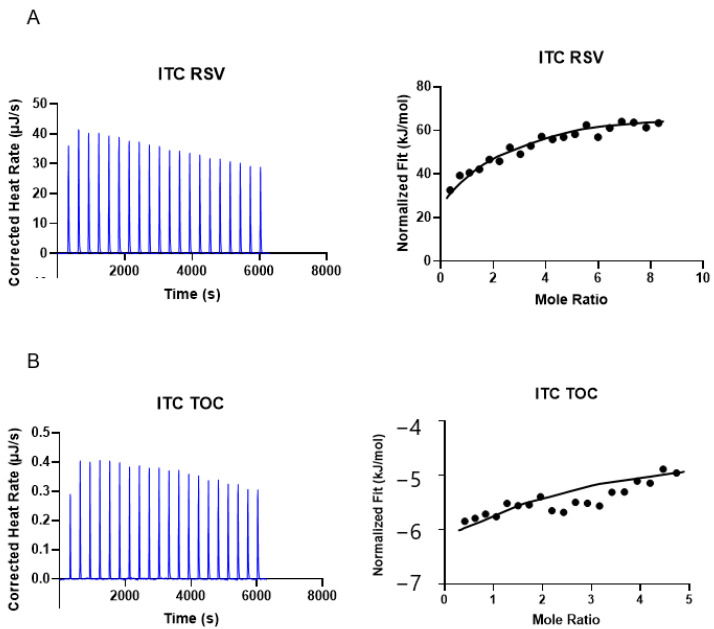
Experimental calorimetric data associated with ITC determinations at 298 K concerning the interactions of 11S with bioactive compounds RSV (**A**) and TOC (**B**), respectively.

The interaction between 11S and RSV or TOC was further examined in detail using ITC. As illustrated in Figure 4 and Table 2, ΔG < 0 and ΔH < 0, indicating that the binding of 11S and TOC or RSV was spontaneous and exothermic, respectively. Additionally, the peaks’ intensity gradually decreased with an increase in the bioactive compound concentration. These findings suggest that 11S globulin gradually saturated, with the binding sites tending to become saturated [11]. In addition, the 11S-RSV had a greater Ka value than 11S-TOC (Table 2). Moreover, the Ka value for 11S-RSV was shown to be one order greater than that for 11S-TOC. This phenomenon would indicate that RSV and 11S globulin had a stronger binding ability, which means that RSV would be more prone to form complexes with this protein.

### 3.5. Fluorescence Spectroscopy

**Figure 5:**
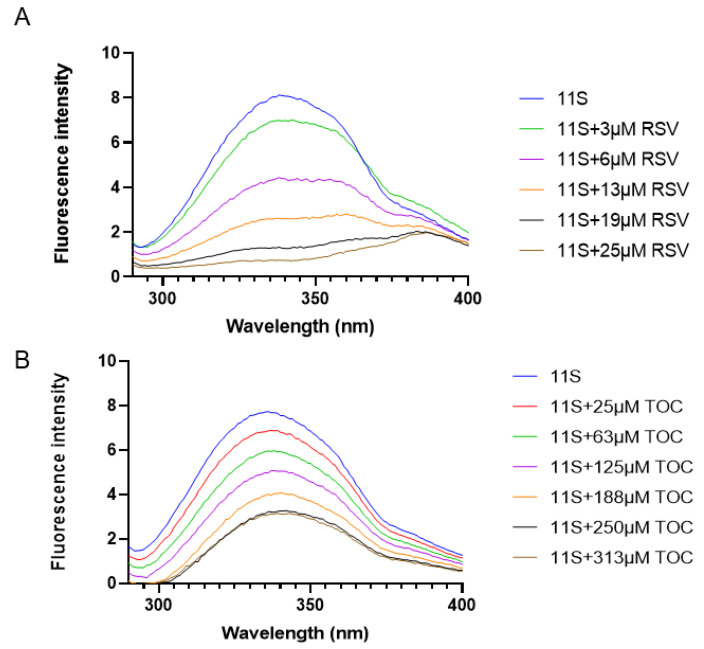
Fluorescence emission spectra of quinoa 11S globulin 0.1%, *w*/*w*, in the presence of various concentrations of RSV (**A**) or TOC (**B**).

Fluorescence spectroscopy is a suitable method for evaluating ligand–protein interactions [8,13]. In this study, interactions between 11S-TOC and 11S-RSV were assessed through their respective fluorescence emission spectra in a mixed system. Figure 5 depicts the emission spectra for the mixed solutions of 11S-TOC and 11S-RSV with varying bioactive compound concentrations, compared to that of the single protein. The maximum fluorescence intensity value was 8.13 at 340 nm with a noticeable shoulder around 380 nm. The quinoa 11S globulin contains four tryptophan residues, and the fluorescence spectrum reflects the contribution of each one. It is well-known that the emission maximum wavelength of tryptophan fluorescence depends on its surrounding environment. In more polar or hydrophilic environments, it emits fluorescence at longer wavelengths [25,37]. Most of the tryptophan residues in this protein are in a hydrophobic environment, leading to a fluorescence emission maximum around 340 nm. However, the shoulder observed in the fluorescence spectrum may be attributed to the contribution of a single tryptophan residue exposed to the solvent.

Upon adding both bioactive compounds, the fluorescence intensity decreased as the TOC or RSV concentration increased in the respective bulk solution. The results on intrinsic protein fluorescence quenching confirm the existence of protein–ligand interactions. To elucidate the type of quenching between the 11S protein and RSV or TOC, the Stern–Volmer and the Scatchard models were applied.

These models were previously used by diverse authors to study the interactions between different proteins and bioactive compounds [8,38,39]. Supplementary Figure S1 shows the dependence of *F*_0_*/F* on RSV or TOC concentration. The value of Ks, the affinity constant, could be obtained from the slope of the curve (Table 3).

When small molecules have the potential to bind independently to a set of equivalent sites on a macromolecule, the equilibrium between free and bound molecules is given by Equation (2). This expression allows us to analyze the dependence of fluorescence intensity on RSV or TOC concentration for static quenching [13]. From the plot *log* [(*F*_0_ *− F*)/*F*] vs. *log* [*BIO*] (Stern–Volmer model), the *n* value can be obtained, which refers to the number of binding sites on the protein molecule. For the Stern–Volmer model, *n* was 1.04 and 2.35 for mixed systems with RSV and TOC, respectively.

Employing the Scatchard model, we obtained a Ks value of 1.5 × 10^5^ M^−1^ for TOC and 3.8 × 10^6^ M^−1^ for RSV, respectively (Table 3). These values manifest the trend observed in the Stern–Volmer model: the binding force of 11S and RSV was one order of magnitude higher than for 11S and TOC. According to the Scatchard model, the parameter *n* was found to be equal to 8.87 and 0.9 for mixed systems with TOC and RSV, respectively (Table 3). The calculated binding sites yield different results when different models are considered, as previously reported [12]. Concerning this, Wei et al. [27], in a very interesting and useful contribution, asserted that researchers could obtain different binding parameters with different mathematical models. So, it is difficult to evaluate which model is more appropriate. This author also obtained different *n* values with the application of different models to the fluorescence data obtained from BSA–RSV mixed systems.

**Figure 6:**
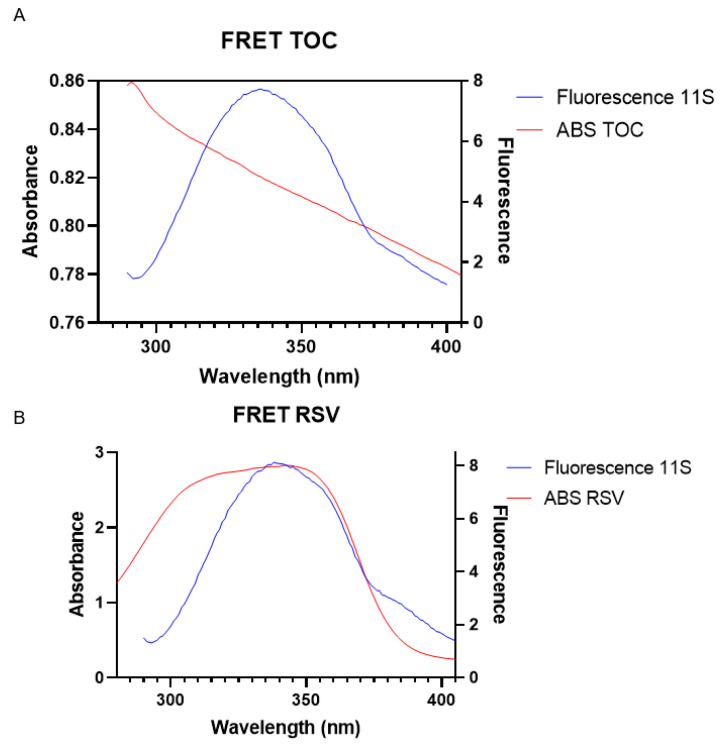
Overlapping between fluorescence emission spectrum of (**A**) 11S and UV absorption spectrum of TOC and (**B**) 11S and UV absorption spectrum of RSV. Both at λex = 280 nm.

Since the fluorescence quenching of 11S by TOC or RSV may make an additional contribution due to Förster resonance energy transfer (FRET), the extension of such a phenomenon was also studied for the whole range of RSV and TOC concentrations. The fluorescence spectrum of 11S globulin and the absorbance spectrum (λex = 280 nm and λem = 290–400 nm) of TOC and RSV overlapped (Figure 6), and the distance (r) between molecules was determined as proposed by Chilom et al. [40].

(7) $$E=1-\frac{F}{F_{0}}=\frac{R_{0}^{6}}{R_{0}^{6}+r^{6}}$$

where *E* is the efficiency of energy transfer, *F*_0_ and *F* are the same parameters as previously described, *r* is the distance between acceptor and donor, and *R*_0_ is the critical distance when the energy transfer efficiency is 50%, calculated as

(8) $$R_{0}^{6}=8.79\times 10^{-5}\times K^{2}\times N^{-4}\times \varphi \times J(\lambda )$$

Here, *K*^2^ is the spatial orientation factor of the donor, *N* is the refractive index of the medium, *Φ* is the fluorescence quantum yield of the donor, and *J*(*λ*) is the overlap integral of the absorption spectrum of the acceptor and the fluorescence emission spectrum of the donor. *J*(*λ*) was calculated with the UV–Vis–IR Spectral Software 1.2, Fluortools software (https://www.fluortools.com/ (accessed on 10 July 2024)) (Table 3). Using *K*^2^ = 2/3, n = 1.336, and *Φ* = 0.118 [40], the critical distance *R*_0_ = 5.39 nm and 6.22 nm was calculated for TOC and RSV, respectively. Additionally, the distance *r* between 11S globulin and TOC or RSV and the efficiency of energy transfer E were determined for each bioactive compound, using Equation (7). As shown in Supplementary Figure S2, the *r* values decreased from 7.79 to 5.09 nm as the TOC concentration varied between 250 and 313 μM. Meanwhile, for RSV, the r values decreased from 15.54 to 4.24 nm, conforming to the RSV concentration, which varied between 1 and 25 μM in the mixed system.

### 3.6. EE Determination

The EE of 11S globulin was in the order of 37 and 73% for RSV and TOC, respectively. The obtained EE values here were higher than those reported by Martinez et al. [12], who achieved an EE of 23% using the purified quinoa 11S globulin for betanin encapsulation.

### 3.7. Analysis of 11S Globulin Aggregation Induced by RSV or TOC

The particle size distribution of 11S and 11S-RSV or 11S-TOC mixed solutions was obtained using DLS. This analysis helps in understanding the aggregation process of proteins, with the aim of either preventing it or leveraging it for specific applications [13]. Numerous investigations have indicated that protein unfolding and aggregation may take place, influenced by factors such as protein composition/concentration, pH, ionic strength, ion concentration, fat content, and other variables [41]. Therefore, the focus was on studying the aggregation of 11S induced by RSV or TOC addition, as this phenomenon may be related to complexation with ligands [42].

**Figure 7:**
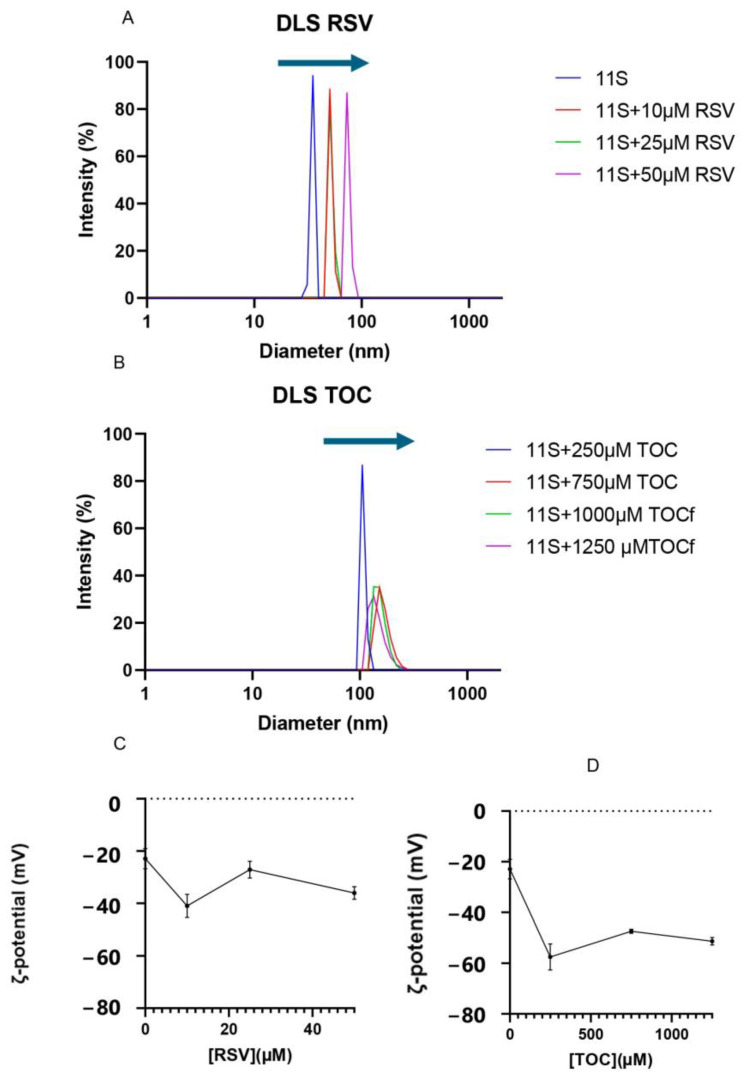
Particle size distribution for 11S-RSV (**A**) and 11S-TOC (**B**) expressed in intensity. Arrows indicate the displacement of the global distribution to higher sizes. Variation of ζ-potential for 11S-RSV (**C**) and for 11S-TOC (**D**), where each point represents the mean ± SD, n = 3.

Intensity vs. particle size distribution analysis showed a single population in all cases (Figure 7A,B). When analyzing the single 11S globulin protein distribution, a single population centered at 31 nm was found. 11S-RSV mixed systems showed an increase in particle size as the concentration of this bioactive increased. At the maximum concentration, a particle reached a mean of 60 nm for the particle size distribution, doubling the size obtained for solutions containing only protein.

Likewise, when TOC was added, an increase in particle size was also observed. In fact, for the two highest concentrations of TOC (1000 and 1250 µM), the particle size could not be measured, as the aggregation induced by this bioactive was extensive and the dimensions of the entities generated exceeded the detection limits of the device. For this reason, it was necessary to filter the samples with a 0.45 µm filter; samples could be read with the equipment, confirming that large aggregates were generated by adding these concentrations of TOC.

ζ-potential emerges as a critical parameter for characterizing nanovehicles, with broad potential applications in industries such as pharmaceuticals, food, nutraceuticals, and cosmetics. The ζ-potential measurements showed that 11S globulin exhibited values of −23 mV (Figure 7C,D), which increased after the addition of bioactive compounds, indicating increased mixture stability. 

### 3.8. Atomic Force Microscopy (AFM)

**Figure 8:**
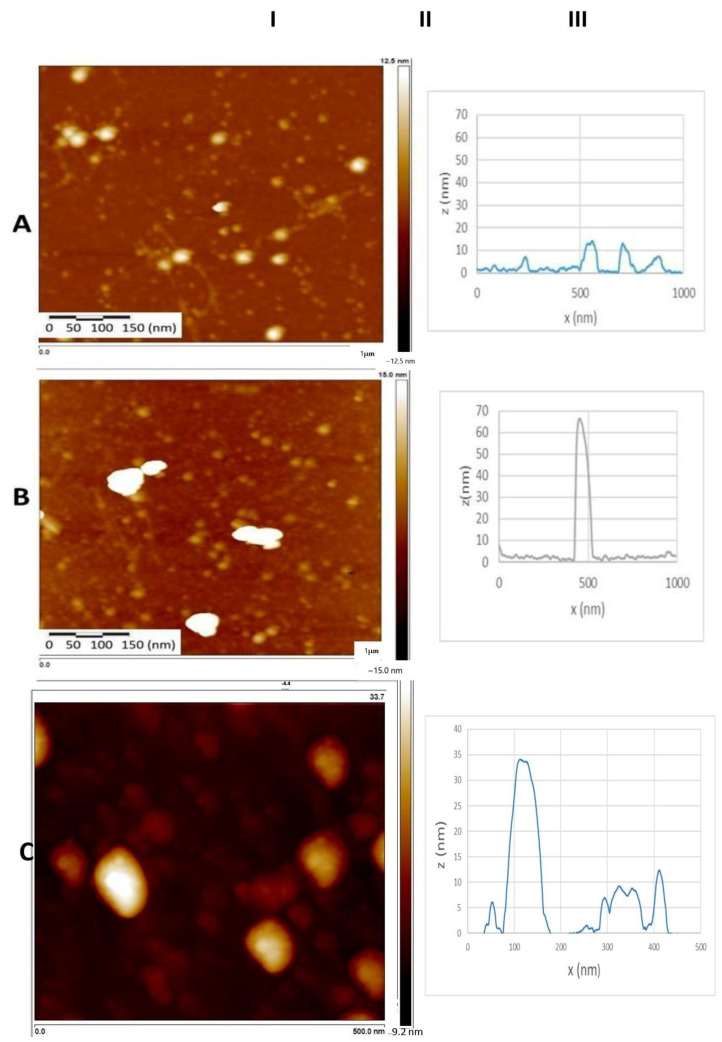
AFM imaging for: (**A**) single 11S globulin; (**B**) 11S+RSV; and (**C**) 11S+TOC mixed systems.

The study of food biopolymers by AFM can be roughly categorized into the provision of new data on commonly used materials, exploring structure–function relationships, and the characterization of newly isolated/synthesized materials [43]. However, only one report was found on the aspects of 11S quinoa-based aggregates using AFM, at least using the most common search engines, which describes the disaggregation induced by the application of high-intensity ultrasound to 11S quinoa aggregates [44]. AFM images are shown in Figure 8, where the analysis for the considered samples is presented (Column I shows the picture with the topographical aspect obtained by the AFM technique; Column II shows the length between the maximum and minimum height possible and Column III describes the height profile, that was taken with a longitudinal section at random across the entire width of the figure (1000 nm)). When taking images of the purified single 11S globulin, particles with an average diameter of 45 nm were observed. RSV and TOC addition also seemed to contribute to a particle size increase, making nanostructures of 100 nm detectable in both cases. This is the confirmation that 11S-RSV and 11S-TOC complexes constituted entities that fell on the nanoscale.

### 3.9. Antioxidant Capacity of 11S-RSV and 11S-TOC Nanocomplexes

#### 3.9.1. ABTS

**Figure 9:**
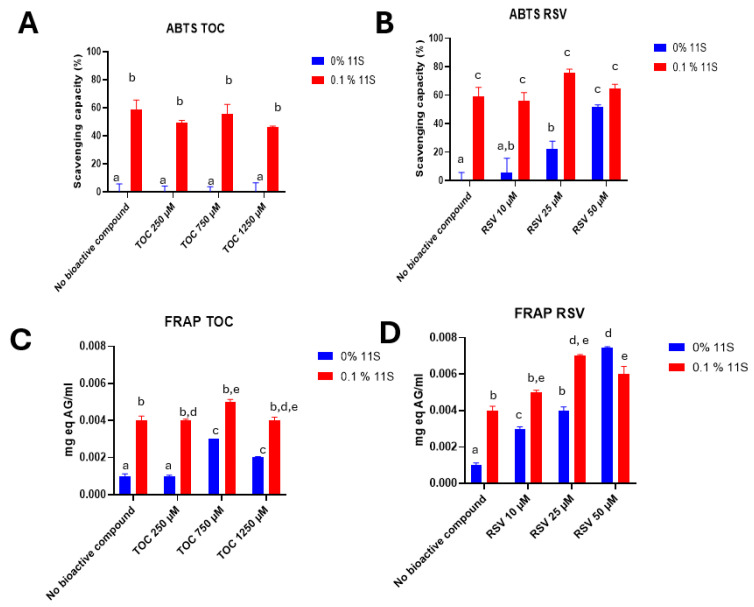
Antioxidant capacity of different concentrations of 11S evaluated by ABTS in TOC (**A**) and RSV (**B**), or FRAP in TOC (**C**) or RSV (**D**) mixed systems. Results are expressed as mean ± SD, n = 3 (*p* < 0.05). Means with the same letter represent non-significant differences (*p* < 0.05).

The ABTS method was used to evaluate the antioxidant capacity of 11S quinoa protein and its mixtures with different concentrations of TOC or RSV (Figure 9A,B, respectively). The protein presented antioxidant capacity on its own; for instance, 11S at the concentration of 0.1% demonstrated an antioxidant activity of 59 ± 6%. This property was previously reported by Martinez et al. [12], and it was attributed to the presence of specific amino acids such as Trp, Tyr and Met, which have the highest antioxidant activity followed by Cys, His and Phe. The antioxidant activities of these amino acids can be explained in terms of their ability to donate hydrogen atoms; Cys can also donate thiol groups [45]. Regarding RSV, as the concentration of this bioactive increased, the antioxidant capacity also increased. RSV’s antioxidant capacity is well known, as widely documented in the literature. For example, Gülçin [46] used a concentration of 10 µg/mL (43µM) of RSV and achieved a near-total decrease in absorbance in the ABTS assay. Concerning TOC, this vitamin did not show any antioxidant capacity, as the bioactive used was in the form of tocopherol acetate. With respect to the mixed systems, the scavenging capacity was equivalent to that of protein alone. This means that the complexing process did not decrease the scavenging capacity of the 11S globulin, which is important.

#### 3.9.2. FRAP

In addition to its anti-radical properties, the antioxidant capability of a bioactive compound can be assessed by its reducing power. This power helps to prevent harmful oxidative reactions by creating a reducing environment. Using the ABTS essay, it was observed that 11S and RSV had antioxidant capacities on their own, whereas TOC did not.

To fully quantify the antioxidant capacity of the produced 11S-RSV or 11S-TOC nanocomplexes, the FRAP assay was also performed. Firstly, a calibration curve was constructed with standard solutions of gallic acid. Then mixtures were made with different concentrations of 11S and RSV or TOC. It can be observed from Figure 9C,D that at null protein concentration, TOC had a greater antioxidant capacity at 750 and 1250 µM. With respect to RSV, the same trend as with ABTS was observed: higher concentrations of RSV correlated with a greater antioxidant capacity. Also, 11S globulin has antioxidant capacity on its own. Concerning this, Escribano et al. [47] also found a great antioxidant capacity in different varieties of quinoa grains by applying the FRAP assay.

## 4. Discussion

The construction of the nanocomplexes, their characterization and the quantification of their antioxidant properties, using detailed techniques, will be analyzed in this section in light of the results obtained, evaluated according to the stated objective, and compared to what other authors have reported. Zhang et al. [48] performed molecular docking between pea 11S protein and the bioactive compounds curcumin, quercetin, and RSV. These authors found binding energies of −5.64, −5.85 and −5.85 kcal/mol, respectively. In this case, RSV bound into a pocket close to TYR161. The docking approach allowed the prediction of the 11S-RSV or 11S-TOC complexes’ formation under specific conditions, and the points of contact on the protein molecule. On the other hand, Chamizo-Gonzalez et al. [49], applying molecular coupling between grape 11S protein and the wine anthocyanin (malvidin 3-O-glucoside), found slightly higher interaction energies than ours, resulting in −7.6 and −8.1 kcal/mol. Patnode et al. [50] also practiced molecular docking between soy 11S globulin and glycerol, sorbitol, or cellulose, finding affinity energies of −4.3, −5.5 and −7.1 kcal/mol, respectively.

According to Chen et al. [51], the modifications in the 11S secondary structure proportions were ascribed to the hydrogen bonding and hydrophobic interaction between 11S globulin and the bioactive compounds, in agreement with the results of interactions predicted by the docking approach. These strong molecular interactions between 11S globulin and the bioactive molecules could induce the partial unfolding and intramolecular reorganization of the protein, leading to the variation of its secondary structure [52].

On the other side, Li and Ni [53] performed ITC for the binding of TOC with trypsin and pepsin. The enthalpy values obtained were −11.37 and −13.78 kJ/mol, respectively, which were shown to be in the same order as that found here for 11S globulin and TOC. Additionally, the Ka was also in the same order (10^3^ M), and the *n* was close to 1. The value of the stoichiometric binding number (*n*) suggests the number of molecules of TOC or RSV combined with one molecule of 11S globulin [53]. Wan et al. [54] worked with 11S of soybeans and found Ka values of the same order (10^3^ M). They considered that a Ka value greater than 10^4^ M would be indicative of high affinity, so the Ka values obtained by Wan et al. [54] and for 11S-TOC in this contribution suggest non-specific interactions. Concerning entropy, the positive value obtained indicates that the interactions had an important entropic component, and in general had predominant hydrophobic interactions, which coincides with what was obtained by the docking analysis for 11S and TOC. On the other hand, the negative ΔS for the 11S-RSV mixed system would imply that the binding is driven by negative enthalpy.

The Kq values were much larger than the maximum admitted for dynamic quenching, which is 1.27 × 10^10^ M^−1^s^−1^ [55]. These results evidence that the fluorescence intensity changed for 11S after TOC or RSV addition, and the effect could be attributed to static quenching, i.e., 11S-RSV and 11S-TOC complex formation. The apparent binding constant Ks was also useful in evaluating the magnitude of molecular interactions. The values reveal a strong binding force between 11S and RSV, which was one order of magnitude higher than for 11S and TOC. Such a result means that the protein manifested a higher affinity for RSV. This result followed the same trend as the results obtained by ITC.

It is interesting to note that the affinity constants obtained through ITC and fluorescence measurements exhibit different values. This discrepancy arises because the measurement principles of both techniques are distinct. In ITC, the constants are derived from thermodynamic equilibria, whereas in fluorescence, the obtained values result from quenching caused by the masking of fluorescent residues due to interactions with the ligand, a photochemical approach.

Regarding FRET analysis, Jiang et al. [56] carried out this analysis between BSA and RSV, finding an r value of 3.47 nm and R_0_ of 2.71 nm, which are lower than those obtained in this work for 11S. These values are much smaller than 7 nm, a criterion value for energy transfer to occur, i.e., the energy transfer from BSA to trans-resveratrol could occur, with a high probability. Therefore, for the 11S globulin, energy transfer would occur for concentrations greater than 125 μM and 6 μM for TOC and RSV.

Concerning EE, and just for comparison, Li et al. [57] designed encapsulated nanoparticles based on a novel construct prepared via the Maillard reaction between a soy protein isolate (SPI) and a polyguluronate, an acidic homopolymer of α-(1,4)-L-guluronate separated from alginate, finding an RSV EE of 86.66%. Khan et al. [58] worked with Kafirin, a prolamin-type protein obtained from sorghum combined with β-lactoglobulin (β-lg) and casein (CAS) for generating nanoparticles via the anti-solvent precipitation method. These entities were used for RSV entrapment, giving an EE of around 73 and 69% for kafirin/CAS and kafirin/β-lg at the maximum RSV concentration used. The ability of pea protein isolates (PPI) to form complex coacervates with different gums (Arabic, tragacanth and tara) was evaluated for the encapsulation of α-TOC by Carpentier et al. [59]. The performance of the complex coacervates was studied according to the protein/polysaccharide mixture and protein/polysaccharide ratio. These authors derived a maximum EE of about 77.4% for the coacervates in comparison with the 53.4% obtained for single PPI. Besides this, Xu et al. [60] developed O/W emulsions with a 5% oil phase and using whey protein isolate–chitosan complexes as emulsifiers in α-TOC encapsulation, at pH 5.7, getting an EE of 86.2%.

The works cited above demonstrate the diversity and complexity of the encapsulation platforms used for RSV and TOC. Thus, it is clear that the EE is highly dependent on the encapsulation platform used. However, in this contribution, the EE values obtained for TOC using a single isolated globulin were in the order of those reported in the literature.

When a bioactive compound is added, different behaviors have been identified in the literature: Ochnio et al. [13] observed an increase in the particle size of soybean 11S globulin after its interaction with folic acid. On the contrary, Penalva et al. [61] reported a drop in the aggregation of casein after its complexation with folic acid and Martinez et al. [12] did not observe changes in the particle size of the 11S globulin of quinoa when adding different concentrations of betanin.

The ζ-potential serves as a measure for the strength of electrostatic interactions among charges at the molecular surface level. Regarding this, the existing literature suggests that electrostatically stabilized hydrocolloids typically possess ζ-potentials surpassing absolute values of 40 mV, as observed by Andreeva et al. [62]. The ζ-potentials obtained here for 11S-TOC nanocomplexes oscillated between −47 and −57 mV. Meanwhilem in the case of 11S-RSV nanocomplexes, ζ–potentials were between −27 and −41 mV. All the examined systems remained colloidally stable, even those with ζ-potential values less than 40. The colloidal stability of these system does not seem to be only rationally explained by electrostatic stabilization, suggesting that other forces can contribute to this effect, stemming from hydrophobic, van der Waals or steric overlapping interactions [62].

Thus, the physical stability does not seem to be rationally explained by electrostatic stabilization, suggesting other forces dictate the system’s stability [15]. A plausible explanation lies in steric overlap interactions, which maintain a separation distance for 11S-RSV and 11S-TOC nanocomplexes, thereby contributing to the stability of the system. These interactions also play a key role in influencing factors such as particle size distribution, cellular uptake, and adsorption to cellular membranes in vivo, as highlighted by Fröhlich [63]. For comparison, it could be mentioned that Corfield et al. [9] and Relkin and Shukat [64] reported ζ-potentials of −23 and −42 mV for solutions of bovine whey protein isolates (WPI) and concentrates (WPC), respectively. These values of ζ-potential, especially the highest (corresponding to WPC), would reflect a greater contribution of electrostatic interactions between complexes made by repulsion, thus increasing the physical stabilization of the dispersions. Corfield et al. [10] and Perez et al. [15] also detected changes in this parameter because of the addition of folic acid (FA) to dairy protein solutions.

Differences in size values obtained from DLS and AFM could be explained in terms of the relationships between sample mounting and the chemical nature of the material under examination. Tiwari et al. [65] attributed such differences to the surface accumulation of protein molecules during sample preparation processes such as spreading and drying, which are very common phenomena. One must keep in mind that samples visualized with AFM lack a hydration layer, which contributes to discrepancies in size. In our case, diameters of 35 and 45 nm were obtained for single 11S by DLS and AFM, respectively. Both types of nanocomplexes, 11S-RSV and 11S-TOC, gave dimensions of 100 nm, as measured by AFM. Meanwhile, hydrodynamic diameters of 72 and 140 nm were registered by DLS for 11S-RSV and 11S-TOC nanocomplexes, respectively.

From the functional point of view, the non-decrease in the antioxidant capacity of nanocomplexes after their formation allowed the single components’ beneficial characteristic to be maintained after complexation, as there are reports indicating that the additive effect in the antioxidant capacity may not be a generality; for example, Rashidinejad et al. [66] observed a drop in the antioxidant capacity of tea polyphenols after their complexation with dairy proteins.

With respect to mixed systems, the trend for 11S-TOC was similar to that for ABTS: the antioxidant activity of the complexes was related to the antioxidant capacity of the 11S globulin. Instead, the effect of combining 11S and RSV was additive. This additive effect implies that the antioxidant capacity of RSV was improved by its complexation with 11S globulin. The term “additive” was once employed to characterize composite systems that exhibited a higher value for a particular physicochemical property compared to the individual components, which attained value did not surpass the sum of the individual components [67]. Based on these results, we attribute the mentioned additive character of the 11S-RSV complexes to the intrinsic antioxidant capacity of 11S globulin.

The global antioxidant power of proteins has been assigned to the antioxidant powers of their constituent amino acids [68,69]. It is likely that the highest concentrations of TOC or RSV be enough to block or mask these amino acids after complexation, and consequently, the antioxidant power of the complexes formed decreased, as determined by FRAP. Protein aggregation induced by complexation with the bioactive compounds cannot be ruled out either, since the highest concentrations of RSV or TOC evidenced this phenomenon through dynamic light scattering, i.e., the displacement of the peaks towards larger sizes (Figure 7A,B).

It is noteworthy that the antioxidant effectiveness of a specific compound may differ across various methods due to factors such as the reaction mechanism employed, the solubility of the antioxidant, the oxidation state, the pH, and the nature of the substrate prone to oxidation. According to this, Biskup et al. [70], who analyzed the antioxidant activity of phenols by both FRAP and ABTS essays, found differences between both techniques. Therefore, it is recommendable to investigate the antioxidant capacity via at least two methods [71].

Mixed systems constituted by 11S-RSV and 11S-TOC exhibited characteristics of ground-state complexes, suggesting their potential application as a delivery system. The obtained results contribute to considering the 11S as a feasible carrier agent for RSV, and as a TOC vehicle and protector.

## 5. Conclusions

In this work, a strategy was developed to design and characterize complexes composed of 11S quinoa seed globulin and RSV or TOC. The particle size distribution allowed us to conclude that these complexes suffered macromolecular aggregation, which was induced after complexation. The dimensions of these complexes/aggregates fell into the nanoscale, constituting nanocomplexes that could serve as vehicles for the bioactive compound, while keeping its beneficial health effects in the presence of light, certain pH and enzymes, during food processing, and during passage through the gastrointestinal tract.

We demonstrated that TOC, the biologically active variant of lipophilic vitamin E, and RSV, a natural polyphenol with antioxidant properties, bound to extracted and purified quinoa seed 11S globulin. Employing different approaches, it can be confirmed that RSV and TOC interacted with 11S in a stable way. Thus, molecular docking, fluorescence analysis, and ITC were employed to gain insights into the molecular interactions between quinoa seed 11S globulin and RSV or TOC. These approaches allow us to obtain affinity constant values and confirm that the interaction between 11S globulin and RSV or TOC primarily involves non-covalent binding. It was observed that the interaction between 11S and RSV is stronger than that between 11S and TOC. Mixed systems of 11S-RSV and 11S-TOC exhibited characteristics of ground-state complexes, suggesting their potential application as a delivery system. The obtained results contribute to considering 11S as a feasible carrier agent for RSV, and as a TOC vehicle and protector. These nanocomplexes could constitute a real platform for the design of functional ingredients and nutraceutical products. This research line will continue with investigations into the behavior of the biological performance of these nanocomplexes, i.e., antioxidant properties, bioaccessibility and bioavailability, as assessed in in vitro systems.

## Figures and Tables

**Table 1:** Parameters derived from molecular docking analysis of 11S-TOC and 11S-RSV mixed systems.

Complex	Binding Energy (Kcal/Mol)	Hydrogen Bonds	Hydrophobic Interactions	Saline Bridge	Total
11S-RSV	−5.6	4	4	0	8
11S-TOC	−6.2	1	10	1	12

**Table 2:** Binding sites (n), binding constant (KA), dissociation constant (Kd), enthalpy change (ΔH), entropy change (ΔS) and Gibbs free energy (ΔG ) for the interaction between TOC or RSV and 11S.

Bioactive Compound	*n*	K_d_ (µM)	K_a_ (M^−1^)	ΔH (kcal/mol)	ΔS (cal/K × mol)	ΔG (kcal/mol)
TOC	0.85 ± 0.03	340 ± 40	(2.9 ± 0.8) × 10^3^	11 ± 1	25 ± 3	4.7 ± 0.2
RSV	0.91 ± 0.04	48.2 ± 1	(2.1 ± 0.3) × 10^4^	10 ± 2	15 ± 1	5.9 ± 0.1

**Table 3:** Fluorescence modeling parameters derived from the application of Stern–Volmer, Scatchard and FRET.

	TOC	RSV
Stern–Volmer model		
Ksv (M^−1^)	4800	385,700
Kq (M^−1^ s^−1^)	1.66 × 10^12^	1.13 × 10^14^
n	1.04	2.35
R^2^	0.9931	0.9406
Scatchard model		
K_s_ (M^−1^)	151,478	3,852,674
n	8.87	0.90
R^2^	0.9422	0.9707
Förster resonance energy transfer (FRET)		
J (λ) (cm^3^/M)	1.14 × 10^13^	2.67 × 10^13^
R0 (nm)	5.39	6.22

## Data Availability

Data will be made available on request.

## Supplementary Materials

The following supporting information can be downloaded at: https://www.mdpi.com/article/10.3390/pharmaceutics16091118/s1, Supplementary Figure S1: Fitting of experimental data with Stern–Volmer (A), Log (B) and Scatchard (C) models for 11S-RSV mixtures. Stern–Volmer (D), Log (E) and Scatchard (F) models for 11S-TOC mixtures. Supplementary Figure S2: Distances between acceptor and donor for 11S globulin and TOC (A) or 11S globulin and RSV (B) derived from FRET analysis
