# Supplementary material for: An Innovative Bio-Vehicle for Resveratrol and Tocopherol Based on Quinoa 11S Globulin—Nanocomplex Design and Characterization
Source: Pharmaceutics. 2024 Aug 24;16(9):1118. doi: 10.3390/pharmaceutics16091118 (PMC11434796; doi:10.3390/pharmaceutics16091118)
Supplement: Supplementary file 1 [file pharmaceutics-16-01118-s001.zip › pharmaceutics-3127034-supplementary.pdf]

Supplementary Materials:

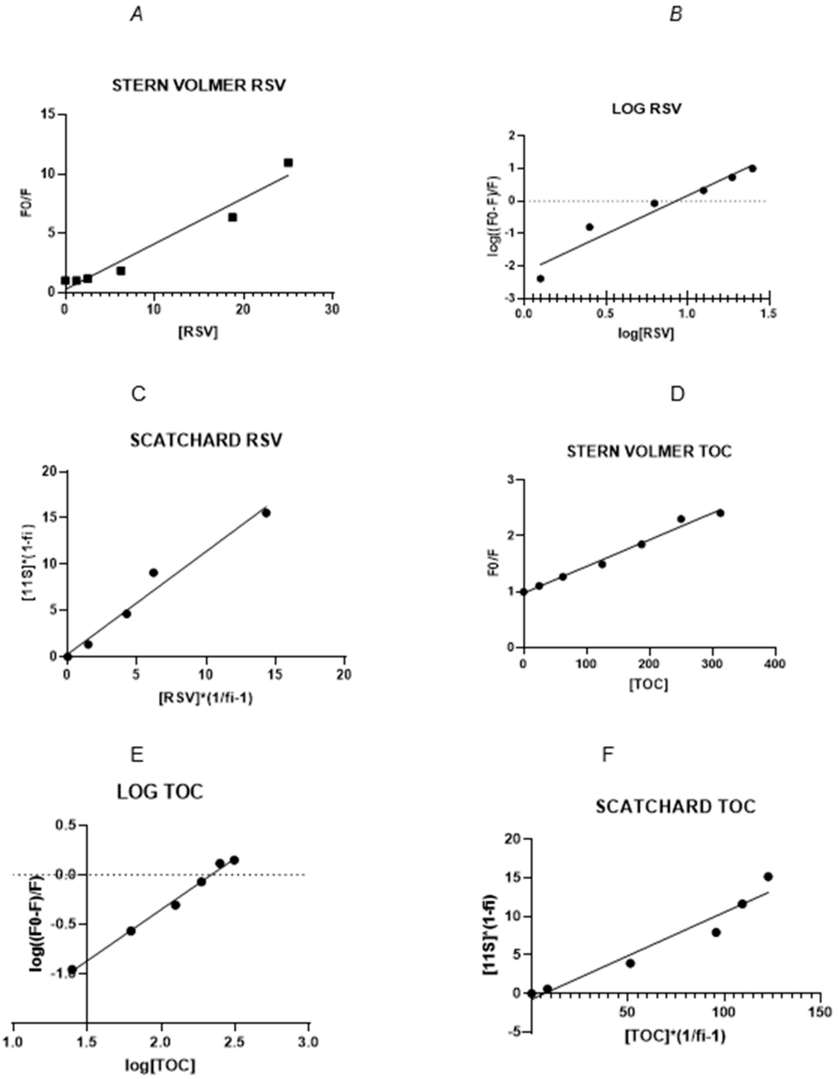

Supplementary Figure 1: Fitting of experimental data with Stern Volmer (A), Log (B) and Scatchard (C) model for 11S-RSV mixtures. Stern Volmer (D), Log (E) and Scatchard (F) model for 11S-TOC mixtures. Dots: experimental data. Continue line: predicted model.

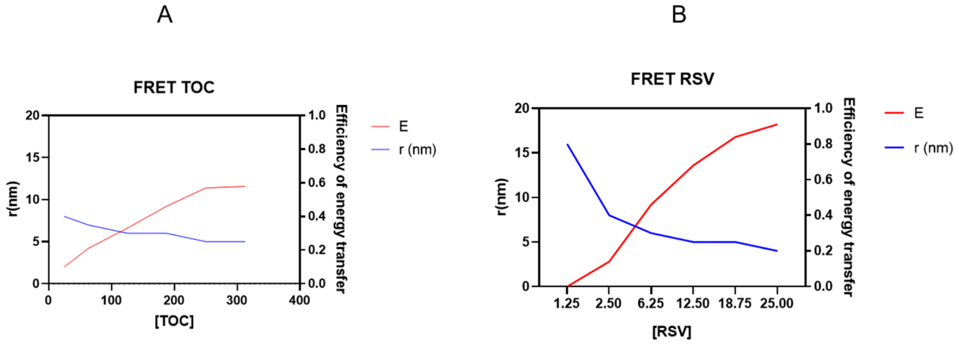

Supplementary Figure 2: Distances between acceptor and donor for 11S globulin and TOC (A) or 11S globulin and RSV (B) derived from FRET analysis.
